# High-Voltage 4H-SiC PiN Diodes: Ion Implantation vs. Epitaxial Growth for Wide-Temperature Operation

**DOI:** 10.3390/ma19040699

**Published:** 2026-02-12

**Authors:** Alfio Samuele Mancuso, Saverio De Luca, Enrico Sangregorio, Annamaria Muoio, Erik Gallo, Silvia Vanellone, Eleonora Quadrivi, Antonio Trotta, Lucia Calcagno, Salvo Tudisco, Francesco La Via

**Affiliations:** 1 Department of Physics and Astronomy, University of Catania, 95123 Catania, Italy; 2 CNR-IMM, VIII Strada n°5, 95121 Catania, Italy; saverio.deluca@cnr.it (S.D.L.); enrico.sangregorio@imm.cnr.it (E.S.); annamaria.muoio@imm.cnr.it (A.M.); 3 Eni S.p.A, Piazzale Mattei 1, 00144 Roma, Italy; erik.gallo@eni.com (E.G.); silvia.vanellone@eni.com (S.V.); eleonora.quadrivi@eni.com (E.Q.); antonio.trotta@eni.com (A.T.); 4 INFN-Laboratori Nazionali del Sud, 95123 Catania, Italy; lucia.calcagno@ct.infn.it (L.C.); tudisco@lns.infn.it (S.T.)

**Keywords:** silicon carbide, p-n junction, high-voltage, high-temperature, deep defects

## Abstract

This study investigates the electrical performance of two 4H-SiC p^+^-i-n^−^ diodes, based on lightly doped epitaxial layers, representative of high-voltage and neutron-detector structures. Each design was implemented in multiple nominally identical devices and characterized over the temperature range 298–623 K, with particular attention to the influence of p^+^ layer fabrication, n-type epitaxial layer thickness, and doping concentration. One diode features an ion-implanted p^+^ layer on a 250 µm thick n-type epitaxial layer, while the other employs an epitaxially grown p^+^ layer on a 100 µm thick n-type epitaxial layer. A comparison of reverse-bias Current–Voltage (I–V) and Capacitance–Voltage (C–V) characteristics indicates that, although both designs exhibit high-quality epitaxial 4H-SiC material, devices with an implanted p^+^ anode tend to show a more pronounced temperature-dependence and degradation of selected electrical parameters in reverse bias than those with an epitaxial p^+^ anode, while forward I–V in the range 298–623 K remains broadly similar for both designs. These observations suggest that anode fabrication and epitaxial design may jointly influence thermal stability, recombination mechanisms, and overall electrical performance, offering guidance for the optimization of 4H-SiC-based power and neutron-detector devices for high-temperature and harsh environments.

## 1. Introduction

The advancement of strategically important technologies in the nanosystem, nuclear engineering, defence, and space sectors necessitates the development of microelectronic components capable of operating under extreme conditions, including high-temperature and intense-radiation environments. Traditional semiconductor materials such as germanium (Ge), silicon (Si) and gallium arsenide (GaAs) are not suitable for these harsh conditions. One promising material for such applications is silicon carbide (SiC). Its bandgap remains sufficiently wide at elevated temperatures, ensuring that thermally generated intrinsic charge carriers are minimal. This characteristic is crucial for maintaining a good performance in high-temperature environments. In addition, SiC material exhibits a high-breakdown electric field (3–6 MV/cm), excellent thermal conductivity (3–5 W/(cm·K)), strong mechanical and chemical robustness, and a relatively high threshold energy for displacement damage (25–35 eV). Together, these electrical, thermal and structural properties underpin the superior resistance radiation hardness of SiC-based devices [1], notably Schottky and p-i-n diodes. For example, R.L.S. Libby et al. demonstrated that SiC Schottky diodes can achieve fast-switching with only a slight increase in the turn-off loss, owing to their reduced forward recovery voltage overshoot [2]. However, the electrical performance of Schottky diodes tends to degrade at high temperatures. The chemical reactions between SiC and refractory metals during fabrication often lead to the formation of undesirable interface states and transition layers, such as unintentionally grown silicon dioxide (SiO_2_), which compromise the thermal stability of the devices [3]. X. Wang et al. investigated the forward Current–Voltage (I–V) characteristics of commercial Schottky diodes between 298 K and 773 K [4]. They observed an increase in forward current density above 773 K, attributed to a reduced electric field in the drift region, while the turn-on voltages decreased from 1 V to 0.8 V due to enhanced thermionic emission. In addition, the on-state resistance increased, leading to higher power dissipation during conduction.

In contrast, S. Shao et al. performed extensive studies combining simulation, fabrication, and characterizations of 4H-SiC p-n diodes [5]. They demonstrated a strong agreement between predictions and experimental results, demonstrating that the turn-on voltage decreases from 2.7 V to 1.45 V as the temperature rises from 290 K to 873 K, confirming that SiC p-n diodes maintain stable performance at high temperatures.

A major technological challenge in the manufacturing of SiC p-n diodes lies in achieving low-resistivity p-type layers. This difficulty arises from the limited solubility of acceptor impurities, the formation of compensating intrinsic defects during equilibrium doping, and the relatively high activation energies of acceptor species [6]. High-temperature ion implantation remains the most effective approach to form highly doped p^+^ regions in 4H–SiC p-n devices, offering excellent spatial selectivity and precise control over the dose and depth profiles. Among available acceptors, aluminum (Al) is preferred to create ion-implanted p–n junctions in SiC due to its good solubility and low ionization energy. However, the electrical activation of Al in 4H-SiC is strongly concentration-dependent. Several coupled physical and electronic mechanisms limit the fraction of implanted atoms that become substitute and ionized acceptors. When the local Al concentration exceeds the solid-solubility limit (~1 × 10^20^ cm^−3^), excess Al tends to cluster or precipitate, becoming electrically inactive [7]. Moreover, higher implantation doses cause greater lattice damage, as energetic ions transfer momentum to lattice atoms through nuclear collisions, creating primary knock-on atoms and displacement cascades. These cascades generate large numbers of Frenkel pairs (vacancies–interstitials pairs), antisites and defect clusters. At high local-damage densities, dislocation loops and amorphous regions can also form, while dynamic recombination during and after implantation leaves behind residual point defects and complexes that introduce deep levels in the bandgap, compensating the intended dopants [8,9].

Once Al atoms occupy substitutional sites and become electrically active, their ability to contribute free holes depends strongly on temperature. Because Al acceptors in 4H-SiC have relatively high ionization energies (~0.17–0.28 eV), thermal ionization is incomplete at low temperatures, leading to the partial freeze-out of holes [10].

Post-implant annealing is therefore essential to enhance Al activation and repair lattice damage. During annealing, wafers are typically encapsulated with a carbon cap to prevent surface degradation [11]. The electrical activation of implanted Al increases with annealing temperature, reaching maximum concentrations around 1 × 10^20^ cm^−3^ in the range of 2223–2373 K and durations between 0.5 and 5 min [12].

However, Rutherford Backscattering Spectrometry/Channelling (RBS/C) and Transmission Electron Microscopy (TEM) studies have shown that, even after optimized annealing, the electrical activation of implanted Al typically remains limited to about 50–60%, although hole concentrations up to 10^18^ cm^−3^ can be achieved [13]. This partial activation is mainly attributed to residual defects that act as deep-level traps or compensating donors, thereby reducing the number of substitutional and ionized Al acceptors, as confirmed by deep-level transient spectroscopy (DLTS) analyses [14]. Only after prolonged annealing, for example 20 min at 2223 K, is near-maximum can Al activation be reached [15], owing to the progressive recovery of implantation-induced damage and the reduction in compensating defects.

To overcome these limitations, epitaxial growth of the Al-doped layer represents an attractive alternative. Pedersen et al. demonstrated that p-type acceptor incorporation during chloride-based chemical vapour deposition (CVD) of 4H-SiC exhibits a pronounced saturation behaviour. Despite increasing the dopant-precursor flow, the net acceptor concentration tends to saturate at values on the order of 10^18^ cm^−3^. This effect is attributed to chlorine-mediated surface reactions combined with thermodynamic and kinetic constraints on dopant incorporation [16]. Although this saturation limits the maximum achievable doping, it also has practical advantages: in situ epitaxial doping yields uniformly substituted, low-defect p-type layers with well-controlled depth profiles and superior crystalline quality, avoiding the lattice damage associated with ion implantation and the need for extreme post-implantation activation anneals.

Consistent with this, Fukaya et al. compared two 4H-SiC p-n diodes in which the p-type layer was formed either by epitaxial growth or Al-ion implantation. The diode with an epitaxially grown p-type layer showed a lower on-resistance, a more uniform distribution of DLTS-detected traps, and consistent cathodoluminescence (CL) signals, providing a clear example of the benefits of chloride-based high-growth-rate CVD for p-type formation [17].

In this work, we investigated the electrical performance of two 4H-SiC p^+^-i-n^−^ diodes over the temperature range of 298–623 K. The first diode (SiC100) features an epitaxially grown p^+^ layer formed on 100 µm thick n-type epitaxial layer. In contrast, the second diode (SiC250) incorporates an ion-implanted p^+^ layer on 250 µm thick n-type epitaxial layer. The SiC250 diode was developed within the Joint-Research-Center CNR-ENI “Ettore Majorana” and reproduces the structural design of a device previously exposed to deuterium–tritium fusion neutrons at the Frascati Neutron Generator (FNG) in Italy [18].

This comparison aims to clarify how the fabrication method of the p^+^ layer, as well as the thickness and doping concentration of the n-type epitaxial layer, influence the electrical characteristics of the devices. The results provide valuable insights for advancing the use of SiC-based detectors in neutron diagnostics for fusion reactors and other demanding environments.

## 2. Manufacturing Device

**Figure 1:**
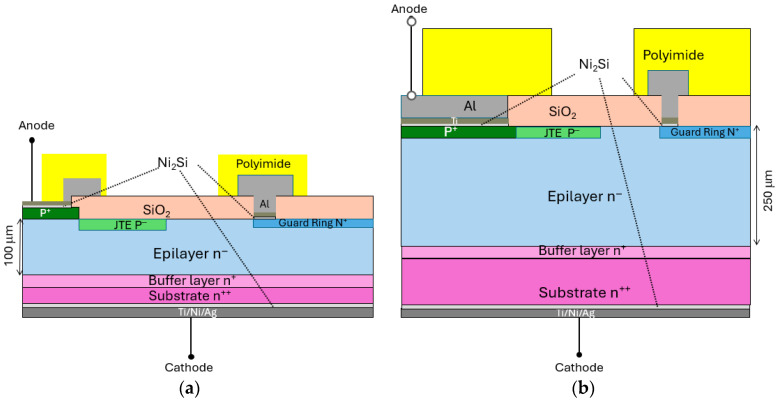
Schematic cross-section of (**a**) SiC100 and (**b**) SiC250 diodes.

The 4H-SiC devices were fabricated at CNR-IMM (Institute for Microelectronics and Microsystems) and INFN-LNS (Laboratori Nazionali del Sud) in Catania. Two device types were produced, with an active thickness of 100 μm and 250 μm and an active area of 25 mm^2^ and 6.25 mm^2^, respectively. The SiC100 diode was fabricated starting from a 100 µm thick n^−^ epitaxial layer grown on a commercially available <0001> 4° off-axis n^++^ 4H-SiC wafer. The epitaxial growth was carried out in an ASM P106 reactor (LPE S.p.A. under ASM International N.V., Catania, Italy) using trichlorosilane (TCS) and ethylene (LPE S.p.A. under ASM International N.V., Catania, Italy) as precursors, with a growth rate of 60 μm/h. Further details on this high growth-rate process can be found in Refs. [19,20]. A 5 μm n^+^ buffer layer was introduced to mitigate the propagation of defects from the substrate into the n-active layer. The p^+^-n^−^ junction was performed by depositing a 0.3 µm thick p^+^ layer with an aluminum acceptor concentration (N_A_) to 1 × 10^18^ cm^−3^ on the n^-^ epilayer, which had a nitrogen donor concentration (N_D_) to 7 × 10^13^ cm^−3^. The edge structures consisted of a p^−^ junction termination extension (JTE) and n^+^ guard rings, obtained by Al^+^ and P^+^ ion implantation, respectively, followed by annealing at 1923 K. An isolation oxide was then deposited, and additional photolithography steps were used to open the contact windows. The front side of the device was metallized by depositing approximately 10 nm of nickel, which was subsequently annealed to form reliable ohmic contacts on both the p^+^ and n^+^ regions through the formation of nickel silicide (Ni_2_Si). After the selective etch removes any unreacted nickel on the oxide, 100 nm thick Ti and 1 µm Al layers were applied along the periphery of the diode for bonding. Finally, a 7 µm thick polyimide layer was deposited to perform the front side passivation. On the backside, the device was mechanically thinned to reduce the total thickness to approximately 110 μm, corresponding to a dead layer of about 10 μm. The backside ohmic contact was then formed by depositing 100 nm of nickel layer and reacting it with the 4H-SiC substrate by laser annealing to form Ni_2_Si. After the silicide formation, a Ti/Ni/Ag metallization stack was deposited on the backside. The SiC250 diode was fabricated using a 250 µm thick n^−^ epitaxial layer on a similar substrate, with the same buffer layer and edge termination design as in the SiC100 diode. The epitaxial growth conditions (growth rate, Si/H_2_ ratio, C/Si ratio, and doping) were identical; only the growth time was increased to reach 250 µm thickness. The main technological difference between the two diodes lies in the formation of the p^+^-n^−^ junction; in SiC250, the p^+^ layer was obtained by ion implantation to achieve a higher acceptor concentration N_A_ to 2 × 10^18^ cm^−3^ on a 250 µm thick active epilayer with N_D_ to 8 × 10^13^ cm^−3^. As for the SiC100 diode, a 7 µm thick polyimide layer was deposited for front-side passivation. No substrate thinning was performed on this diode; however, the same backside metallization process was used. A schematic cross-section scheme of the two diode structures is reported in Figure 1.

## 3. Experimental Setup

The devices were tested by using a Nextron probe station (Nextron Co., Ltd, Busan, South Corea) interfaced with a Keithley 237 High Voltage Source-Measure Unit (SMU) (Keithley Instruments, LLC, Solon, OH, USA) for Current–Voltage characteristics (I–V) and model 4200-SCS for Capacitance–Voltage (C–V) characteristics. The temperature was varied from 298 to 623 K with a step of 25 K under nitrogen flow. At each step of the temperature, the forward I–V characteristics were measured by sweeping the DC bias from 0 to 5 V in steps of 25 mV, with a compliance current of 10 mA. The reverse characteristics were measured by sweeping the DC bias from 0 to −1000 V in steps of −5 V, with a compliance current of 1 μA. Every 50 K, the C–V characteristics were acquired using the ACS Basic software version 2.1 by sweeping the DC bias from 0 to −200 V in steps of –1 V.

Both I–V and C–V measurements were performed by recording a single value at each temperature in the range 298–623 K; therefore, statistical error bars from repeated measurements are not available. The accuracy of the source-measure unit and the stability of the probe-station contacts were verified experimentally to ensure repeatable measurements. On this basis, we estimate that the uncertainty of each data point is smaller than the symbol size in the plots and does not affect the extracted parameters discussed in the following sections. In addition, we have characterized several nominally identical devices of each type with the same active area; all of them exhibit very similar I–V and C–V characteristics, with extracted parameters agreeing within the experimental uncertainty, indicating that the measurements are reproducible across multiple diodes.

## 4. Results

### 4.1. I–V Characteristics

**Figure 2:**
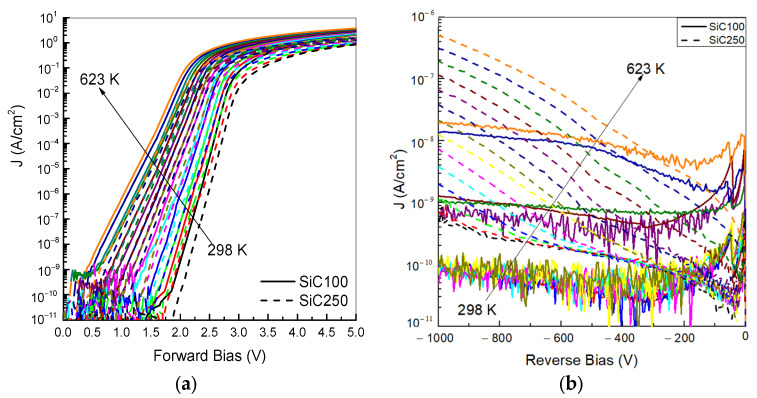
(**a**) Forward J–V and (**b**) reverse J–V curves at different temperatures ranging from 298 K to 623 K in steps of 25 K. The curves are normalized for the area of the different devices. The color code corresponds to 298 K (black), 323 K (red), 348 K (green), 373 K (blue), 398 K (cyan), 423 K (magenta), 448 K (dark yellow), 473 K (navy), 498 K (purple), 523 K (wine), 548 K (olive green), 573 K (dark cyan), 598 K (royal blue), and 623 K (orange).

The measured forward J–V curves of two SiC diodes are depicted in Figure 2a. In the voltage range 2.0–2.5 V, the total forward current density (J_tot_) is dominated by diffusion–recombination processes. At a higher forward bias, the increase in J_tot_ becomes less steep due to the onset of series resistance effects. As the temperature increases, J_tot_ at given forward voltage V_F_ rises because of an enhanced thermal carrier diffusion and recombination rate. Consequently, the forward J–V curves of both diodes shift towards a lower bias [21]. At the same time, the effective series resistance increases at high temperatures.

The diodes were also characterized under reverse bias in the temperature range 298–623 K and for reverse biassing from 0 to −1000 V, as shown in Figure 2b. At −1000 V, the SiC100 exhibits a lower reverse leakage current density of 8 × 10^−11^ A/cm^2^ at 298 K, which increases only slightly to 1.53 × 10^−10^ A/cm^2^ at 498 K. This weak apparent temperature-dependence is largely attributed to measurement, due to the limited current sensitivity of the SMU (≈1 × 10^−11^ A). Above 498 K, the leakage current starts to increase more markedly, reaching 7.27 × 10^−10^ A/cm^2^ at 523 K, and 2.04 × 10^−8^ A/cm^2^ at 623 K. In this temperature range, the leakage current is mainly driven by thermally assisted carrier generation through deep levels in the bandgap [22,23].

Over the temperature range, the SiC250 exhibits a higher leakage current density to 4.81 × 10^−10^ A/cm^2^ at 298 K, and a more pronounced curvature in the reverse J–V characteristics. This leakage in this device is likely related to a poorer insulation oxide quality. In particular, the post-oxidation anneals in oxygen (POA), which typically improve oxide stoichiometry and densification and reduces oxygen vacancies, were omitted. The lack of this step can result in a non-stoichiometric, more porous oxide structure with a higher density of structural defects and interface traps [24,25], thereby facilitating leakage conduction through defect-assisted tunnelling. At an elevated temperature, the leakage current density further increases, reaching 5.03 × 10^−7^ A cm^−2^ at 623 K under −1000 V.

### 4.2. Capacitance–Voltage Characteristics

**Figure 3:**
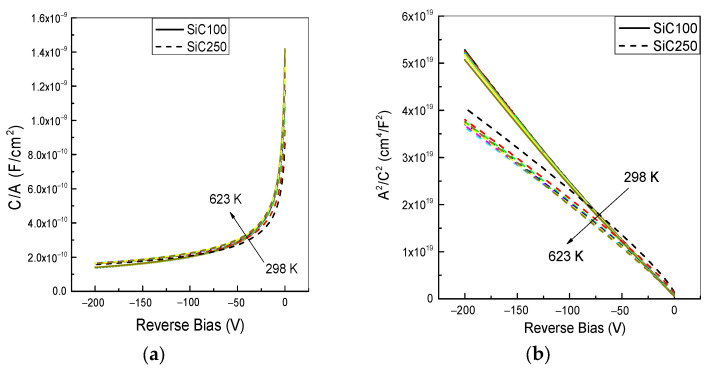
(**a**) C–V characteristics, (**b**) Reciprocal of the squared capacitance, in the temperature range 298–623 K, with a temperature step of 50 K. The curves are normalized for the device area. The color code corresponds to 298 K (black), 323 K (red), 373 K (green), 423 K (blue), 473 K (cyan), 523 K (magenta), 573 K (yellow) and 623 K (dark yellow).

As shown in Figure 3a, the C–V characteristics of the SiC100 diode remain essentially unchanged over the temperature range 298–623 K. The corresponding 1/C^2^–V curves exhibit a good linear behaviour at all investigated temperatures, as depicted in Figure 3b. This weak temperature-dependence indicates a stable depletion charge and junction capacitance throughout the probed temperature range.

In contrast, the SiC250 diode exhibits an excess capacitance and convex $1/C^{2}$–V dependence. Such behaviour is consistent with spatial dopant inhomogeneity in the implanted p^+^ layer, which modifies the bias dependence of the depletion width. In addition, carrier exchange with interface traps located at the SiO_2_/4H-SiC interface can introduce an extra capacitance contribution whose magnitude varies with temperature [26].

## 5. Discussion

### 5.1. I–V Analysis

**Figure 4:**
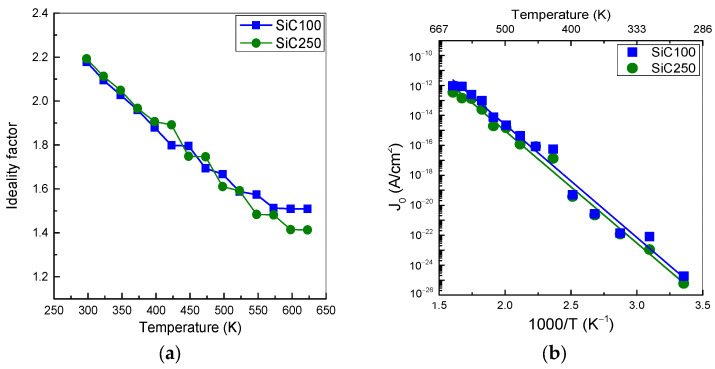
(**a**) Thermal distribution of ideality factor and (**b**) Arrhenius plot of J_0_ in the temperature range 298–623 K. The estimated uncertainty of each point is smaller than the symbol size (also valid for the other figures).

The measured forward J–V curves of two SiC diodes in the temperature range 298–623 K are depicted in Figure 1a. The total current density J_tot_ is described by compact analytical Equation (1) in the form of

(1) $$J_{\mathrm{tot}}\approx \;J_{0}{\;e}^{\frac{q\;(\left|V_{\mathrm{bi}}\;-\;V_{F}\right|\;-\;IR_{s})\;}{n\;k\;T}}\;$$

where $J_{0}$ is the saturation current density, q is the electric charge, V_F_ is the applied forward bias, V_bi_ is the built-in voltage, R_s_ is the series resistance, k is the Boltzmann constant, and *n* is the ideality factor [27,28]. Considering the series resistance contribution in the diffusion–recombination region irrelevant, we have extrapolated n and J_0_ parameters from the theoretical fit of the experimental data. At room temperature, SiC100 exhibits an ideality factor of 2.18, consistent with the presence of generation–recombination centres in the junction region [29]. The ideality factor decreases to 1.51 to 623 K, as shown in Figure 4a. The SiC250 diode exhibits an ideality factor of 2.19 at RT, which relates to a higher density of defects in the thicker epitaxial layer. The ideality factor decreases rapidly to 1.41 at 623 K. On the other hand, a relevant change in pre-exponential parameter J_o_ is noticeable through the Arrhenius diagram, as depicted in Figure 4b. The calculated activation energy is (1.50 ± 0.07) eV for SiC100 and 1.48 ± 0.06 eV for SiC250 in the range 298–623 K. These values closely agree with each other and fall within the range typically associated with SRH recombination–diffusion in 4H-SiC pn junctions, suggesting that both diode designs are governed by comparable dominant recombination processes under forward bias [27,29].

Furthermore, we have calculated the activation energy ΔE to investigate the behaviour of the reverse leakage current by the following Equation (2):

(2) $$J_{rev}\;\propto e^{-\frac{Ea}{kT}}$$

**Figure 5:**
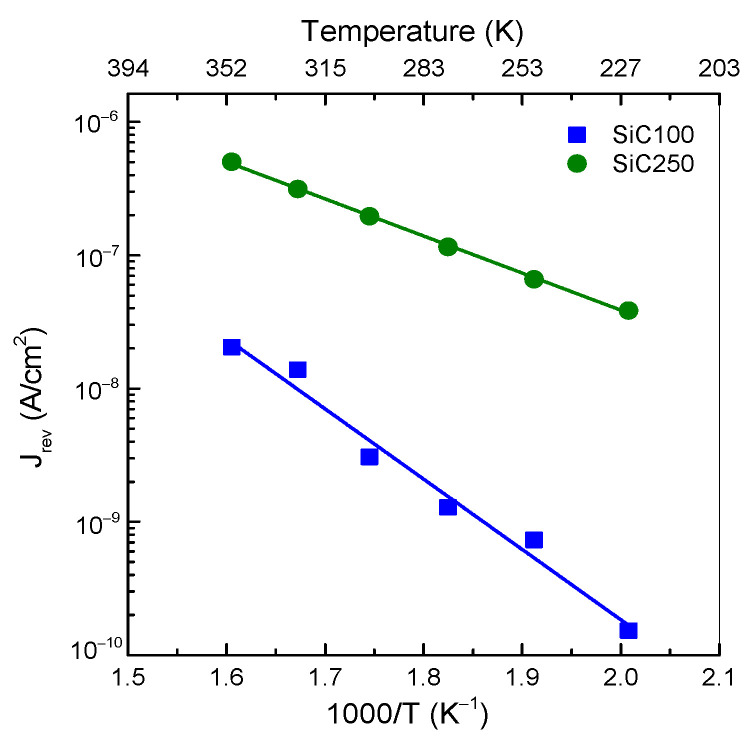
Arrhenius plot of reverse leakage current in the temperature range 498–623 K.

For the analysis of the temperature dependence of the leakage current, we consider a reverse bias of –1000 V to study the temperature dependence of leakage behaviour, as shown in Figure 5. In the SiC100 diode, the angular coefficient of the linear fit yields an activation energy of (1.05 ± 0.07 eV), which is much lower than the 4H-SiC bandgap (E_g_ = 3.26 eV). This suggests that the $J_{rev}$ is not governed by generation–recombination (typically E_a_ ≈ E_g_/2) nor by Auger or radiative recombination (E_a_ ≈ E_g_) [30,31]. Instead, the leakage current is primarily influenced by tunnelling effects.

At −1000 V, thermofield emission becomes the dominant transport mechanism: a fraction of the carriers can tunnel directly through the potential barrier under the influence of the electric field, resulting in an increase in leakage current up to 2.04 × 10^−8^ A/cm^2^ at 623 K. In addition, when the applied voltage is not yet sufficient to enable full direct tunnelling, trap-assisted tunnelling can occur via deep levels located below the conduction band. The extrapolated activation energy is close to 1 eV, consistent with the EH_4/5_ defect levels located about 1 eV below the conduction band. These centres are generally associated with the (+/0) charge-state transition of the carbon antisite–vacancy defect (CAV). In DLTS, this transition appears as a deep electron trap: the positively charged CAV^+^ captures an electron during the filling pulse to form CAV^0^, and then thermally re-emits it into the conduction band (CAV^0^ → CAV^+^ + e^−^), with a typical activation energy of ≈1 eV [32,33].

In this temperature range, the SiC250 diode exhibits higher leakage current density and a more pronounced curvature of J–V characteristics compared to the SiC100 diode, but the lower activation energy to (0.55 ± 0.01 eV) again suggests that J_rev_ is governed by tunnelling mechanisms rather than traditional generation–recombination processes. This activation energy is consistent with the Z_1/2_ centre, previously observed in our DLTS study on a similarly fabricated SiC250 detector [34] and commonly associated with acceptor states of the carbon vacancy (V_C_). This behaviour can be rationalized by assuming two concurrent contributions to the reverse leakage current: a field-controlled component associated with the poorer insulating oxide quality and related interface states, which remain essentially constant at a fixed reverse bias, and a temperature-dependent component linked to the Z_1/2_ deep level, as reflected by the extracted activation energy.

### 5.2. Thermal Built-In Potential Distributions

When an external forward bias V_F_ is applied to the 4H-SiC diodes, the effective barrier potential that normally opposes carrier flow is reduced. As V_F_ increases, the total barrier across the junction decreases to V_bi_ − V_F_, where V_bi_ is the built-in potential. This reduction facilitates carrier transport across the junction, resulting in an increased forward current. When the current is dominated by diffusion and recombination processes, V_bi_ can be estimated using Equation (3):

(3) $$V_{\mathrm{bi}}\;=\;V_{F}\;+\;\frac{k\;T}{n\;q}\;\ln\left(\frac{J_{\mathrm{tot}}}{J_{o}}\right)\;$$

**Figure 6:**
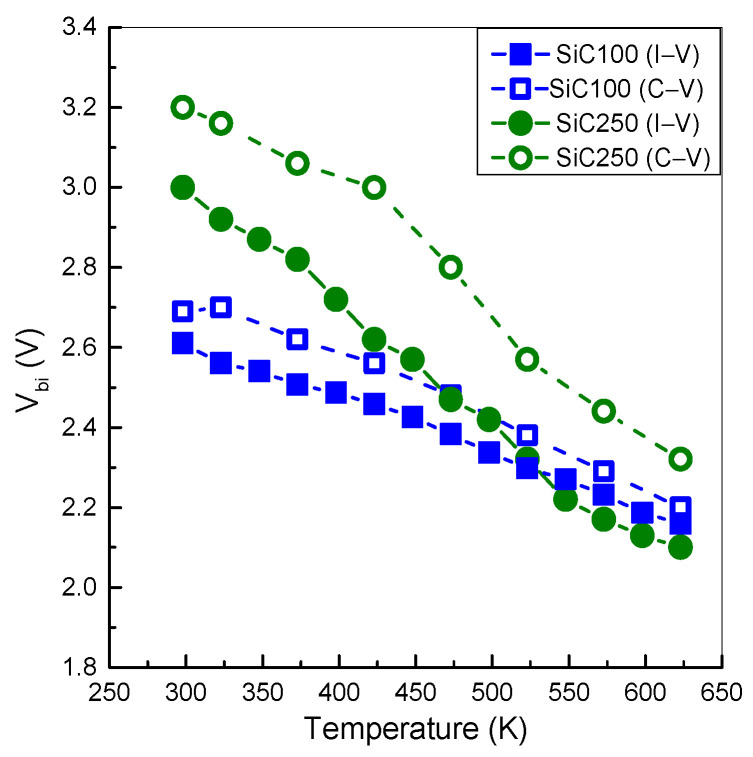
Built-in potential distributions in temperature range 298–623 K for SiC100 and SiC250. Solid lines and open symbols refer to V_bi_ values calculated by C–V characteristics, while dash lines and closed symbols to V_bi_ values extrapolated by forward I–V characteristics.

The built-in potentials V_bi_ extracted for SiC100 and SiC250 are reported in Figure 6. As the temperature increases, both diodes show the expected decrease in V_bi_, consistent with the rise in the intrinsic carrier concentration. For SiC100, V_bi_ decreases from about 2.6 V at 298 K to 2.16 V at 623 K. The lower V_bi_ and its comparatively weaker temperature-dependence are consistent with the lower p^+^ doping, which reduces the junction space-charge and effective barrier height [35]. In contrast, SiC250 exhibits a higher V_bi_ of about 3.0 V at 298 K and a more pronounced temperature-dependence, dropping to approximately 2.1 V at 623 K. This enhanced sensitivity to temperature may be related to a higher effective p^+^ doping and to implantation-induced non-uniformities in the junction region, which could modify the carrier recombination and perturb the equilibrium potential.

Complementary values of V_bi_ are also extrapolated from the Capacitance–Voltage (C–V) characteristics using Equation (4):

(4) $$V_{\mathrm{bi}}\;=\;\frac{\mathrm{kT}}{q}\;+\;\ln\left(\frac{N_{A}\;N_{D}}{n_{i}^{2}}\right),$$

which yields slightly higher V_bi_ values than those obtained from I–V analysis. At room temperature, SiC100 shows a V_bi_ value of 2.6 V, which decreases to 2.2 V at 623 K (see Figure 6). Both SiC diodes show an apparent bandgap narrowing with increasing doping in the p-n junctions, leading to a reduction in V_bi_ at higher temperatures [36,37].

The observed difference between V_bi_ values derived from I–V and C–V measurements stems from the distinct measurement conditions. The I–V characteristics are influenced by recombination and series resistance under non-equilibrium conditions. In contrast, C–V measurements are performed near equilibrium, thus providing a more accurate estimation of V_bi_ values [38].

### 5.3. Temperature Dependence of Dopant Activation

When a reverse bias V_R_ is applied, the differential capacitance associated with the depletion region in the drift layer is related to the applied voltage, as expressed in Equation (5):

(5) $$\frac{1}{C^{2}}=\;\frac{2\;(V_{R}-V_{\mathrm{bi}})}{A^{2}\varepsilon_{0}\varepsilon_{S}q\;N_{D}}$$

where $\varepsilon_{0}$ is the vacuum permittivity, $\varepsilon_{S}$ is the relative dielectric constant of 4H-SiC.

**Figure 7:**
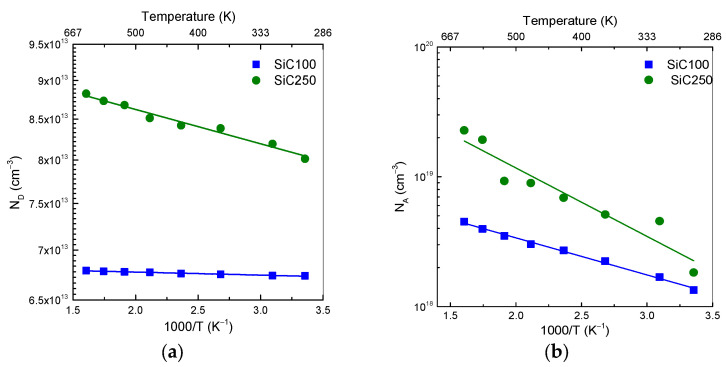
Arrhenius plots of (**a**) donor concentration for nitrogen-doped (n-type) and (**b**) acceptor concentration for aluminum-doped (p^+^-type) 4H-SiC.

The N_D_ values for both diodes are extracted from the slope of the 1/C^2^–V plots. For SiC100, the n^−^ epilayer exhibits a very low N_D_ value of 6.73 × 10^13^ cm^−3^ at 298 K, increasing only slightly to 6.79 × 10^13^ cm^−3^ at 623 K, as depicted in Figure 7a. The SiC250 diode instead shows a higher N_D_ of 8 × 10^13^ cm^−3^ at RT, rising to 8.8 × 10^13^ cm^−3^ at 623 K. From the temperature dependence of N_D_, extremely small activation energies to (0.39 ± 0.02 meV) are obtained for SiC100.

Previous studies report that the ionization energy of nitrogen (N) donors is approximately 61 meV, and so almost all N donors are already ionized at 298 K [6,30]. In lightly doped regions, the Fermi level lies well-below the conduction band edge and shifts slightly toward the midgap as the intrinsic carrier increases with the temperature. This shift reduces the effective activation barrier (E_C_ − E_F_), partially compensating the increased thermal energy and leading to a nearly temperature-independent free-electron density [1]. Similarity, the N-doped ionization energy of (40.04 ± 0.26 meV) justifies a weak temperature-dependence of free-electron density due to the low N_D_ concentration in SiC250 [39].

Using the built-in potential V_bi_ and the doping concentration of the epitaxial layer extracted from the C–V measurements, the temperature dependence of the acceptor density N_A_ (T) was determined, as shown in Figure 7b. The SiC100 diode exhibits a N_A_ value of 1.32 × 10^18^ cm^−3^ at RT, rising to 4.50 × 10^18^ cm^−3^ at 623 K. On the other hand, SiC250 exhibits a higher N_A_ value of 1.83 × 10^18^ cm^−3^ to 298 K, increasing to 2.28 × 10^19^ cm^−3^ at 623 K, indicating a stronger temperature-dependence. From the Arrhenius plot, the acceptor ionization energy value of (54.87 ± 1.27 meV) for the SiC100 diode is lower than the extrapolated value of (104.65 ± 12.93 meV) for SiC250. The difference between these values is strongly influenced by the uniformity and substitutional incorporation of the Al dopants. The energy level calculated in SiC100 is comparable to the first excited states of Al acceptors in SiC, as predicted by the hydrogenic model [40,41]. At doping levels of approximately 10^18^ cm^−3^, the Coulomb potentials of ionized Al impurities overlap, leading to random local potential fluctuations. These fluctuations distort the valence-band edge, generating an exponential tail of localized states that extend into the bandgap. By introducing localized tail states near the valence-band edge, the band tail provides intermediate states into which holes can be thermally excited, thereby reducing the effective energy required to ionize Al acceptors [42,43]. In the SiC250 diode, the situation is more complex. The Al acceptors are introduced by implantation, and residual implantation damage can favour the formation of Al-related defect complexes. These complexes may alter the local potential landscape and shift the effective acceptor level, leading to the higher apparent ionization energy extracted from the temperature dependence of N_A_ [44].

### 5.4. Temperature Dependence of Carrier Mobilities

**Figure 8:**
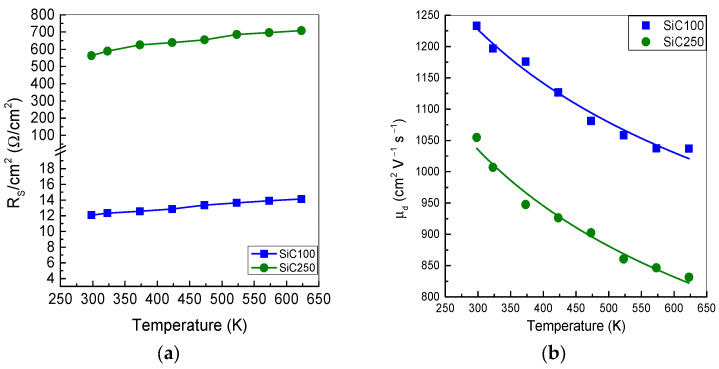
Series resistance (**a**) and drift mobility (**b**) in the range 298–623 K.

The total series resistance R_S_, given by the sum of the bulk, contact, and material resistance, the contact resistance, and n^-^ epilayer contributions, is calculated from the slope of the forward J–V characteristics in the high-bias (quasi-ohmic) region, and normalized for the different area of the two detectors. For very thick epilayers, the dominant contribution to R_S_ comes from the drift layer, so the substrate and contact resistances can be neglected. At room temperature, the series resistance per unit area is 12.07 Ω/cm^2^ for SiC100, lower than the value of 565.6 Ω/cm^2^ measured for SiC250, consistent with the higher resistance of the thicker epilayer, as shown in Figure 8a. In both devices, R_s_ increase over the temperature range 298–623 K, mainly due to the reduction in electron drift mobility (µ_d_) with temperature, while N_D_ remains nearly constant [45].

The drift mobility µ_d_ can be estimated from the series resistance using Equation (6):

(6) $$\mu_{d}=\;\frac{L}{qN_{D}R_{s}A}$$

where L is the thickness of n^−^ region and A is the cross-sectional area of the diode.

As shown in Figure 8a, SiC100 exhibits $\mu_{d}$ value of 1233 cm^2^ V^−1^ s^−1^ at RT, which is higher than the value of 1020 cm^2^ V^−1^ s^−1^ predicted in the Caughey–Thomas (C–T) model at the corresponding doping level [46]. In this diode, the nitrogen donor ionization energy is low enough that all N atoms are ionized already at RT, and the total density of the ionized scattering centre is smaller than assumed in the C–T model, resulting in a somewhat-higher mobility. As the temperature is increased to 623 K, phonon scattering becomes more pronounced, and $\mu_{d}$ decreases to about 1040 cm^2^ V^−1^ s^−1^.

Similarly, the SiC250 exhibits a lower $\mu_{d}$ of 1050 cm^2^ V^−1^ s^−1^ at RT, consistent with the higher N_D_ value measured from C–V characterization (Figure 7a), and $\mu_{d}$ further decreases to about 830 cm^2^ V^−1^ s^−1^ at 623 K due to the combined effects of ionized-impurity and phonon scattering.

The drift mobility is fitted as function of temperature by using a power–law relationship (7):

(7) $$\mu_{d\;}≅\;T^{-\beta },$$

where β is the temperature exponent, which depends on factors such as the doping concentration and crystallographic direction. F. La Via et al. reported that the parameter β was approximately equal to three for an n-type doping concentration of 1 × 10^16^ cm^−3^ due to phonon scattering, which leads to a stronger temperature-dependence [47]. In contrast, the exponent extracted from our data are significantly smaller, β = 0.2 for SiC100 and β = 0.3 for SiC250, indicating a much weaker decrease in µ_d_ with temperature. This behaviour is consistent with the very-low nitrogen doping levels and the nearly complete donor ionization in our epilayers, so that mobility is governed by a balance between ionized-impurity and phonon scattering rather than by phonons alone over the investigated temperature range.

### 5.5. Temperature Behaviour of Drift Collection Time

When a p–n junction is subjected to reverse bias, the depletion region (W) widens, establishing V_bi_ that governs charge carrier motion within this space-charge region [48]. Assuming an abrupt, one-sided junction with uniform doping on the depleted side, W is expressed either from the measured capacitance or from the voltage-based expression, by following Equation (8):

(8) $$W\;=\;\frac{\varepsilon_{S}\;\varepsilon_{0}A}{C}\;=\;\sqrt{\left[\left(V_{\mathrm{bi}}-V\;-\frac{2\;k\;T}{q}\right)\frac{2}{{q\;\varepsilon }_{s}{\varepsilon_{0}N}_{D}}\right]}\;$$

**Figure 9:**
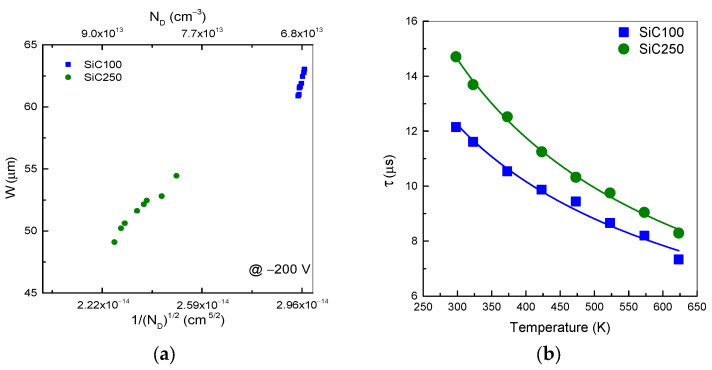
(**a**) Depletion width as a function of (1/N_D_) ^1/2^ (bottom x-axis) extrapolated at −200 V. The top x-axis reports the corresponding N_D_. obtained by inverse transformation of the bottom axis. All data points belong to the same dataset. (**b**) diffusion time in the temperature range 298–623 K.

At −200 V, the lightly N-doped SiC100 diode exhibits the largest W value of 63 µm at RT, as depicted in Figure 9a. At 623 K, the increase in the intrinsic carrier generation reduces $V_{bi}$, resulting in a smaller W of approximately 61 µm. In contrast, the SiC250 diode, more heavily doped on the n-side, shows a narrower W of about 54 µm at RT. This reduction follows directly from the inverse square-root dependence of W on N_D_. A higher donor-density provides a sufficient charge balance over a shorter distance, reducing the depletion width required to sustain V_bi_. Raising the temperature to 623 K, the reduction in V_bi_ led to the narrowing of W to 49 µm.

Under reverse bias and low-injection conditions, the injected minority carrier density remains significantly lower than the equilibrium majority carrier concentration. Under a low-injection condition, the generation and recombination of the minority carriers dominate the transient behaviour [49,50]. In this regime, the Hall mobility (µ_h_) is related to the drift mobility through the Hall scattering factor (γ_H_), as described by Equation (9):

(9) $${µ}_{h}\;=\;\gamma_{h}\;{µ}_{d}$$

Considering the N_A_ value to 1.2 × 10^18^ cm^−3^, the thermal distribution of $\gamma_{H}$ was taken from the study of S. Asada et al. in the temperature range 298–623 K [51].

The drift collection time of minority carriers (τ) across the depletion region is given by Equation (10) below:

(10) $$\tau =\;\frac{q\;W^{2}}{\mu_{h}k\;T}$$

The temperature dependence of the drift collection time for the two 4H-SiC pn diodes is shown in Figure 9b. For the SiC100 diode, the collection time decreases from approximately12.7 µs at 298 K to 7 µs at 623 K. This trend reflects the temperature dependence of the Hall mobility and of the internal field: at a fixed bias, the drift time scales approximately as τ ∝ 1/μ_h_. The continuous reduction in t_drift_ with an increasing temperature therefore indicates an overall increase in the effective carrier velocity in this temperature range, consistent with a progressive weakening of ionized-impurity scattering and a moderate impact of phonon scattering up to 623 K [52]. In contrast, the SiC250 diode exhibits a systematically lower Hall mobility and, correspondingly, a longer drift collection time from 15 µs at 298 K to 8 µs at 623 K, which suggests a higher density of charged defects and/or stronger compensation in this sample. A full separation of the individual scattering contributions is beyond the scope of this work, but the correlation between Hall mobility and drift collection time is clear in both devices.

## 6. Conclusions

In this study, we investigated the electrical performance of two 4H-SiC p^+^-i-n^−^ diodes, incorporating a p^+^ layer formed either by epitaxial growth (SiC100) or by Al-ion implantation (SiC250), over the temperature range 298–623 K. The electrical behaviour of each design was found to be consistent and reproducible across multiple nominally identical devices. Under forward bias, the evolution of the ideality factor and the extracted activation energies for both diodes fall within the range typically associated with deep-level generation–recombination processes in 4H-SiC p^+^-i-n^−^ junctions and are therefore consistent with a significant SRH mechanism, although the extracted mobilities confirm the high quality of the epitaxial layers in both designs.

SiC100 exhibits a lower built-in potential and a comparatively weaker temperature-dependence of key parameters, indicating improved thermal stability within the investigated range. By contrast, SiC250 shows a higher built-in potential and a more pronounced temperature sensitivity. This behaviour correlates with features in the C–V and reverse-bias characteristics that are compatible with enhanced interface-trap activity and junction non-uniformity in the implanted structure. At −1000 V reverse bias, leakage in both diodes is dominated by a tunnelling mechanism: SiC100 shows an activation energy of ≈1.05 eV, associated with EH_4/5_, while SiC250 shows ≈0.55 eV, attributed to Z_1/2_ centres.

Although the p^+^ epitaxial layer in SiC100 has a relatively low doping concentration (≈1 × 10^18^ cm^−3^), its electrical characteristics are significantly less temperature-dependent than those of the implanted diode (SiC250). These results show that the method used to fabricate the anode (epitaxial grow versus ion implantation) can have a stronger impact on high-temperature stability than modest differences in doping, providing concrete guidance for the design of SiC devices intended for harsh environments.

A more systematic study, in which (i) diodes with different thicknesses are fabricated using the same technology and (ii) diodes of different technologies are realized with the same thickness, will be required to fully decouple these contributions, and we identify this as an important direction for future work.

## Data Availability

The original contributions presented in this study are included in the article. Further inquiries can be directed to the corresponding authors.
